# Associations of parental feeding practices with children’s eating behaviors and food preferences: a Chinese cross-sectional study

**DOI:** 10.1186/s12887-023-03848-y

**Published:** 2023-02-18

**Authors:** Chao Qiu, Rosalind Hatton, Qian Li, Jiale Xv, Jiaqin Li, Jiahe Tian, Shenghao Yuan, Min Hou

**Affiliations:** 1 College of Humanities, Jiangnan University, 1800 Lihu Road, Wuxi, China; 2 Bradford District Care NHS Foundation Trust, Bradford, UK; 3 School of Public Health, College of Medicine, Shanghai Jiao Tong University, 227 Chongqing South Road, Shanghai, China

**Keywords:** Children, Eating behavior, Food preference, Parental feeding practice

## Abstract

**Background:**

Childhood inadequate eating behaviors contribute to the epidemic of obesity. Previous research suggests that parental feeding practices are partially associated with development of eating behaviors among children, but the results are inconsistent. The present study was to investigate whether parental feeding practices were associated with eating behaviors and food preferences among Chinese children.

**Methods:**

A cross-sectional study was conducted to collect data from 242 children (ages 7–12) in six-primary schools in Shanghai, China. A series of questionnaires including parental feeding practices and children’s eating behaviors have been validated, and were completed by one of parent who has responded for child’s daily diet and living. In addition, researchers instructed children to complete the questionnaire of food preference. After adjustment for children’s age, sex and BMI status, as well as parental education and family income, the linear regression analysis was used to evaluate relationships of parental feeding practices with children’s eating behaviors and food preferences.

**Results:**

Parents with boys had higher level of control overeating practice than those with girls. Mothers who responded to child’s daily diet and living and completed feeding practices questionnaire used a greater level of emotional feeding practices than fathers. Boys had higher levels of food responsiveness, emotional overeating, enjoyment of food and desire to drink than girls. Boys had different preferences for meat, processed meat products, fast foods, dairy foods, eggs, and snacks and starchy staples & beans from girls. In addition, scores of instrumental feeding practice and preference for meat significantly differed among children with different weight status. Furthermore, parental emotional feeding practice was positively associated with children’s emotional undereating (β 0.54, 95% CI 0.16 to 0.92). There were also positive associations of parental encouragement to eat with children’s preference for the processed meat (β 0.43, 95% CI 0.08 to 0.77). Moreover, instrumental feeding practice was negatively associated with children’s fish liking (β -0.47, 95% CI -0.94 to -0.01).

**Conclusion:**

The current findings support associations of emotional feeding practice with some children’s emotional undereating, as well as parental encouragement to eat and instrumental feeding practice related to preference for processed meat and fish, respectively. Further studies should continue to ascertain these associations using longitudinal designs, and to evaluate efficacy of parental feeding practices impacting developments of healthy eating behaviors and preferences for healthy foods among children by interventional studies.

**Supplementary Information:**

The online version contains supplementary material available at 10.1186/s12887-023-03848-y.

## Background

The prevalence of overweight and obesity among children has remarkably increased across the world [1, 2]. Numbers of overweight and obese children has a tenfold increase over the last 30 years in China [3]. Childhood obesity is associated with a wide range of health problems, such as hypertension, dyslipidemia, insulin resistance, dysglycemia, fatty liver disease and psychosocial complications [2]. This is likely to continue into adulthood [4]. Thus, there is a clear need to understand etiology of obesity and make strategies to prevent from obesity among children.

The development of obesity is influenced by genetical and environmental factors [1]. It is suggested that understanding childhood eating behavior is essential to develop strategies to reduce prevalence of obesity [5]. The most commonly used tool to define children’s eating behaviors is the Child Eating Behavior Questionnaire (CEBQ) involving eight items [6, 7]. Generally, these eight items of eating behaviors can be divided into two main dimensions: food approach (food responsiveness, emotional overeating, enjoyment of food and desire to drink), and food avoidance (satiety responsiveness, slowness in eating, emotional undereating and food fussiness) [6, 7]. Jansen and her colleagues conducted a cross-sectional research which reveals that children with higher level of food responsiveness and enjoyment of food tended to have greater body mass index (BMI), whilst emotional undereating, satiety responsiveness and food fussiness were negatively linked to children’s BMI [8]. A prospective cohort study found a bi-directional positive association between emotional overeating and BMI among middle-class Dutch children [9]. Power et al. also presented a longitudinal bidirectional relationship between emotional overeating and weight status among Hispanic children at eight-years-old [10]. In addition, body weight was positively associated with score of food responsiveness, but negatively related to score of satiety responsiveness in children aged 4 years [10]. Previous studies suggested that higher weight status is associated with higher scores on food approach, but related to lower scores on food avoidance [11–13].

It is also known that childhood obesity is partially attributed to unhealthy eating patterns [2]. Consumptions of fruits, vegetables, pulses and nuts were negatively associated with BMI measurements among children (6–7 years) and adolescents (13–14 years) across countries [14]. However, a descriptive study has shown a negative association between frequency of meat intake and overweight and obesity among children and adolescents [15]. In addition, consumption of sweetened beverages was associated with risk of overweight or obese among Australian children [16]. Food preference is associated with food consumption [17–19]. Previous studies demonstrated that the liking for fats or sweet foods was positively associated with weight status among children [20, 21].

Wardle and her colleagues suggested that parental feeding practice might contribute to the emergence of differences in children’s weight [22]. Parental feeding practices are also associated with children’s eating behaviors. For example, parental emotional feeding practice was positively associated with emotional overeating in children aged 8 to 12 years [23, 24]. A cross-sectional study showed that maternal controlling feeding is positively linked to emotional over- and under- eating among children aged 2 to 5 years, while paternal controlling feeding is associated with children’s slow eating and emotional undereating in the UK [25]. In addition, it has been found that children aged 5–7 years with parents likely using food as a reward and restriction of food, tended to develop emotional overeating behavior about two year later [26].

On the other hand, maternal pressure to eat is negatively associated with consumption of fruits and vegetables among children aged 2- to 6-year old in London [27]. In addition, children with parents using encouragement to eat and controlling over eating behaviors likely consumed more fruits and vegetables [28]. Conversely, preschool children in Hong Kong whose parent highly used instrumental and emotional feeding practices tended to eat less fruits and vegetables [28]. However, studies examining parental feeding practices in relation to food preferences are relatively sparse. previous research demonstrated that Dutch children at 6–7 years whose parents were likely to use encouragement feeding practice had less snacking preference [29].

A clear understanding of parental feeding practices in relation to children’s eating behaviors and food preferences has important implications for educators, parents and clinicians to foster child’s healthy eating behaviors. However, there is a lack of information on associations between parental feeding practices and children’s eating behaviors and food preferences among Chinese children. Therefore, in this study we aimed to conduct a cross sectional study to evaluate the associations of parental feeding practices with child’s eating behaviors, and to assess the associations between parental feeding practices and child’s preferences for foods in China.

## Methods

### Study design

This cross-sectional study collected data from school-children aged 7–12 years in six primary schools including public and private schools in Shanghai, China in 2019. Those without serious organ disease, abnormal physical development or physical impairments were included in this study. Schools were randomly selected from the districts with upper, moderate, and lower levels of socioeconomic status using a stratified random sampling method. Children were randomly selected from each grade within the schools using random numbers via computer systems. Their parents were informed by class teachers, and those who consented were recruited. The questionnaire included the information on participant’s characteristics, parental feeding practices and children’s eating behaviors and food preferences. The questionnaires of participant’s characteristics, parental feeding practices and children’s eating behaviors were completed and sealed in the envelop by child’s mother or father who has responded for child’s daily diet and living, and then was brought to the researchers by their children. The researchers in the classroom guided children to complete questions on preferences for all of food items. Ethics approval was obtained from the Ethics Committee of School of Public Health of Shanghai Jiao Tong University for Human Subject Research, and all the parents gave written informed consent.

### Participant’s characteristics

Parents reported children’s sex and age. Children’s weight and height were obtained from annual school health records. The underweight, normal-weight, overweight and obesity were categorized based on the sex- and age- specific BMI cutoff points established by the Working Group for Obesity in China (WGOC) [30], which has been explored to be consistent with Eastern Asia ethnic characteristics of body fatness growth [31]. The WGOC of the International Life Science determined cutoff points of underweight, overweight and obesity of children and adolescents aged between 7- and 18-years old by using smoothening, fitting and graduating on the B-spline curve and established the BMI reference norm with age and sex for screening underweight, overweight and obesity officially [30]. Furthermore, parents were required to self-report his or her sex, education and family income. Parental education was categorized as ‘Secondary high school or lower’, ‘High school or equivalent’, and ‘Bachelor’s degree or equivalent’ and ‘Master’s degree and above’. Annual family income was categorized as ‘less than RMB 80,000’, ‘less than RMB 80,001–150,000’, ‘RMB 150,001–300,000’ and ‘more than RMB 300,000’ based on national family income classes reported by central bank of China.

### Parenting feeding practices

The parental feeding practices were measured with the well-established parental feeding practice questionnaire (PFPQ) [22], which is available and used to assess feeding practices for parents whose children were 6–12 years old [29, 32, 33], and has been translated and validated into the Chinese version [34]. The questionnaire includes 27 items with response options categorized across five levels, namely: ‘never’, ‘rarely’, ‘sometimes’, ‘often’ and ‘always’. This questionnaire assesses four aspects of parental feeding practices including ‘Instrumental Feeding (i.e. rewarding or punishing children with food to modify the child’s behavior [35])’, ‘Emotional Feeding (i.e. using food to influence the child’s emotions [36])’, ‘Encouragement to eat (i.e. encouragement of the child’s interest and curiosity to taste and eat a variety of foods [37])’ and ‘Control over Eating (control on food accessibility and moments of eating [37] via 4, 5, 8 and 12 questions, respectively. Cronbach alpha’s was 0.65 for encouragement to eat, 0.67 for control over eating, 0.60 for instrumental feeding and 0.77 for emotional feeding.

### Children’s eating behaviors

The Children’s Eating Behavior Questionnaire is a multi-dimensional, parent-reported questionnaire [38], which has been translated and validated into Chinese version [39]. Parent who was responsible for child’s daily diet and living was required to complete the questionnaire. It consists of 35 items with response options categorized across five levels, namely ‘never’, ‘rarely’, ‘sometimes’, ‘often’ and ‘always’, representing eight eating behaviors, including ‘enjoyment of food (4 items)’, ‘food responsiveness (5 items)’, ‘emotional overeating (4 items)’, ‘desire to drink (3 items)’, ‘satiety responsiveness (9 items)’, ‘slowness in eating (9 items)’, ‘emotional undereating (4 items)’ and ‘food fussiness (6 items)’[38]. The questionnaire has good internal consistency, concurrent validity with actual eating behavior, test–retest reliability, and stability over time [40, 41]. In present study, Cronbach alpha was 0.79 for desire to drink, 0.70 for slowness in eating, 0.88 for food responsiveness, 0.94 for satiety responsiveness, 0.73 for emotional undereating, 0.68 for emotional overeating, 0.82 for food fussiness, 0.90 for enjoyment of food.

### Food preferences

As no validated measure of food preferences was available, the authors modified the food preference questionnaire from Fildes’ study [42]. The questionnaire contains 79 food items covering foods that has been widely consumed, according to food availability in Chinese market and data of dietary intake for Chinese children [43]. For example, ‘Turkey’ was removed because of non-traditional Chinese food. ‘Green bean’, ‘White turnip’, ‘Chinese watermelon’ and ‘Pineapple’ were selected due to foods in abundant supply. Moreover, ‘Cheese (processed)’, ‘Cheese (hard)’ and ‘Cheese (cream)’ have been combined into ‘Cheese’ because of limited types available in China. Trained researchers instructed children to subjectively consider each food item with 6 response options as follows: ‘never tried’, ‘dislikes a lot’, ‘dislikes’, ‘neither dislikes nor likes’, ‘likes’ and ‘likes a lot’, and to make an assessment as to whether or not they would like to eat it by responding one of options, and limited their thinking time for any one of the food items. ‘Never tried’ was recorded as missing, and ‘dislikes a lot’, ‘dislikes’, ‘neither dislikes nor likes’, ‘likes’ and ‘likes a lot’ was scored as -2, -1, 0, 1 or 2, respectively. Those foods were excluded from analyses if they were reported as being ‘never tried’ by more than 75% of children. Food items were categorized based on food group and process, such as ‘vegetables’, ‘fruits’, ‘meats’, ‘fish’, ‘processed meat products’, ‘fast food’, ‘dairy foods’, ‘eggs’, ‘snacks’, ‘starchy staples and beans’ (Table S1), which were scored as the liking for food groups. Cronbach’s alpha for these 10 food-group scales showed an acceptable internal reliability, which was 0.71 for dairy foods, 0.92 for fruits, 0.85 for meats, 0.65 for fish, 0.67 for processed meat products, 0.69 for fast food, 0.58 for eggs, 0.87 for snacks, starchy staples and beans for 0.84, and 0.93 for vegetables.

### Statistical analysis

We obtained means and standard deviations (SD) for continuous variables: age, weight, height, scores of parental feeding practices, children’s eating behaviors and food preferences, and frequencies and percentages for categorical variables: sex, BMI status, parental education and family income. The normal distribution was determined by the Kolmogorov–Smirnov test. To examine differences among groups, nonparametric tests with Mann–Whitney U or Kruskal–Wallis one-way ANOVA were used for non-normally distributed variables. Variance inflation factors (VIF) were analyzed to address potential multicollinearity for linear regression models. VIF for predictor variables such as parental feeding practices were under 2, indicating no major multicollinearity issues. Covariates included in the fully adjusted models were those known from previous literature to have an association with children’s eating behaviors and food preferences or those found to have association on univariable analyses. Covariates for consideration in the fully adjusted models included children’s age, sex and BMI category, parental education and family income. In linear regression models, after the adjustment for potential confounding covariables, we estimated β-coefficient with 95%CI to investigate parental feeding practices in relation to children’ eating behaviors, and to assess associations between parental feeding practices and children’ food preferences. As there were too few parents whose education level were lower than secondary high school (2.9%), or higher than master’s degree (4.5%), we combined secondary high school or lower and high school or equivalency (12.7%) into one category, and combined bachelor degree or equivalency and master’s degree or above into another category for the linear regression analyses. In addition, family income was categorized into ≤ 150,000 RMB and > 150,000 RMB due to few households with incomes less than 80,000 RMB or more than 300,000 RMB. Analyses were completed with the IBM SPSS program, version 22 (IBM, Chicago, IL, USA). Two-sided *P* < 0.05 was considered to be statistically significant.

## Results

### Participants’ characteristics

In total, we recruited 242 children (131 boys and 110 girls, one was missing) with a mean age of 9.0(1.5) years. Children’s age, sex, weight, height, and BMI, parental sex, education, family income, scores of parental feeding practices, children’s eating behaviors and food preferences are shown in Table 1. The prevalence of being underweight, overweight, and obese among children were 7.5%, 17.8%, and 8.7% respectively. The most parents (88.0%) obtained a bachelor’s degree or above, and 66.4% of families had an annual income more than 150,000 RMB.

**Table 1:** Participants’ characteristics

Characteristics		N (%)	Mean ± SD
Age			9.0 ± 1.5
Sex	Male	131(54.6)	
	Female	110(45.4)	
Weight (kg)			33.25 ± 11.16
Height (cm)			137.19 ± 11.36
Child’s BMI status	Underweight	18(7.5)	
	Normal weight	160(66.0)	
	Overweight	43(17.8)	
	Obese	21(8.7)	
Parent’s sex	Male	77(31.9)	
	Female	165(68.1)	
Parent’s education	Secondary high school or lower	7(2.9)	
	High school or equivalency	15(6.2)	
	Bachelor degree or equivalency	202(83.5)	
	Master’s degree or above	11(4.5)	
Household income, RMB	< 80,000	10(4.3)	
	80,000 ~ 150,000	71(29.4)	
	150,001 ~ 300,000	135(55.7)	
	> 300,000	26(10.7)	

Mean ± SD reported for continuous variables

The use of encouragement to eat by parents was the greatest, followed by emotional feeding practice, instrumental feeding practice and control over eating (Table 2). The score of enjoyment of food among children was the highest, followed by emotional undereating, food responsiveness, and food fussiness (Table 2). Among 79 food items, the only food that had been tried by less than 75% of children was canned fish (55%) (Table S1). The most children did not like pepper and bacon (Table S1). Twenty-one foods scored more than one point, which were in fruit group (*n* = 11), fast-food group (*n* = 1), dairy food group (*n* = 1), snacks group (*n* = 5), and starchy foods and beans group (*n* = 3). Fruits were the most liked foods by children, followed by snacks (Table 2).

**Table 2:** Scores of parental feeding practices, children’s eating behaviors and food preferences

		**Mean ± SD**
Parental feeding practices	Instrumental feeding	2.41 ± 0.69
	Encouragement	3.28 ± 0.57
	Emotional feeding	2.49 ± 0.73
	Control over eating	0.63 ± 0.49
Children eating behavior	Food responsiveness	2.59 ± 0.92
	Emotional overeating	2.22 ± 0.95
	Emotional undereating	2.74 ± 0.89
	Enjoyment of food	3.19 ± 0.95
	Desire to drink	2.53 ± 1.03
	Satiety responsiveness	1.61 ± 0.78
	Slowness in eating	1.34 ± 0.87
	Food fussiness	-0.18 ± 0.80
Food preference	Vegetables	0.55 ± 0.71
	Fruits	1.15 ± 0.58
	Meats	0.55 ± 0.85
	Processed meat products	0.53 ± 0.91
	Fast foods	0.68 ± 0.88
	Dairy foods	0.93 ± 0.80
	Eggs	0.68 ± 1.12
	Snacks	0.99 ± 0.71
	Starchy staples & Beans	0.71 ± 0.70

Mean ± SD reported for continuous variables

### Differences in parental feeding practices, children’s eating behaviors and food preferences

Parental feeding practices and children’s eating behaviors were not significantly different with children's age from 7 to 12 years old (all *P* > 0.050, Figure S1 and S2). As shown in Table 3, parents who have boys highly used control over eating practice than those having girls (*P* = 0.042). No difference was found in other items of parental feeding practices (*P* > 0.050). In terms of eating behaviors, boys had higher levels of food responsiveness, emotional overeating, enjoyment of food and desire to drink than girls (all *P* < 0.050). In addition, there were significant sex differences in enjoyment of food, emotional overeating, desire to drink, food responsiveness and slowness in eating among older children (11–12 years old), but not younger children (7–10 years old) (Figure S3). Furthermore, preferences for meat, processed meat products, fast foods, dairy foods, eggs, and snacks and starchy staples & beans were different between boys and girls (all *P* < 0.050).

**Table 3:** Differences in parental feeding practices and children’s eating behaviors according to children’s sex and weight status

	**Sex** ^a^			**Weight status** ^b^				
	**Boys**	**Girls**	***P* value**	Under weight	Normal weight	Overweight	Obesity	***P* value**
**Parental Feeding Practice**								
Instrumental feeding	2.43 ± 0.70	2.38 ± 0.70	0.581	**2.17 ± 0.50**	**2.36 ± 0.73**	**2.51 ± 0.65**	**2.76 ± 0.65**	**0.033**
Encouragement	3.27 ± 0.55	3.29 ± 0.59	0.884	3.30 ± 0.60	3.31 ± 0.56	3.18 ± 0.48	3.24 ± 0.70	0.701
Emotional feeding	2.44 ± 0.68	2.55 ± 0.77	0.234	2.34 ± 0.60	2.48 ± 0.74	2.48 ± 0.79	2.74 ± 0.73	0.516
Control over eating	**0.70 ± 0.44**	**0.55 ± 0.53**	**0.042**	0.66 ± 0.46	0.65 ± 0.50	0.63 ± 0.48	0.54 ± 0.50	0.844
**Children’s Eating Behavior**								
Food responsiveness ^a^	**2.71 ± 0.91**	**2.43 ± 0.92**	**0.013**	2.65 ± 0.87	2.57 ± 0.95	2.51 ± 0.90	2.77 ± 0.75	0.564
Emotional overeating ^a^	**2.39 ± 0.97**	**2.01 ± 0.89**	**0.001**	2.10 ± 1.11	2.22 ± 0.94	2.07 ± 1.05	2.43 ± 0.81	0.267
Emotional undereating	2.84 ± 0.92	2.61 ± 0.83	0.083	2.93 ± 0.95	2.69 ± 0.85	2.88 ± 1.14	2.71 ± 0.79	0.650
Enjoyment of food ^a^	**3.40 ± 0.92**	**2.91 ± 0.92**	** < 0.001**	3.31 ± 0.90	3.13 ± 0.92	3.29 ± 1.21	3.36 ± 0.84	0.425
Desire to drink ^a^	**2.71 ± 1.02**	**2.29 ± 0.99**	**0.001**	2.60 ± 1.05	2.48 ± 1.06	2.64 ± 0.98	2.68 ± 0.80	0.398
Satiety responsiveness	1.61 ± 0.81	1.62 ± 0.74	0.848	1.49 ± 1.02	1.61 ± 0.77	1.71 ± 0.81	1.56 ± 0.63	0.866
Slowness in eating	1.26 ± 0.89	1.47 ± 0.83	0.141	1.51 ± 1.18	1.33 ± 0.82	1.38 ± 0.95	1.30 ± 0.84	0.895
Food fussiness	-0.11 ± 0.80	-0.27 ± 0.77	0.080	-0.12 ± 0.89	-0.22 ± 0.79	-0.30 ± 0.89	0.14 ± 0.61	0.178
**Food Preference**								
Vegetables	0.61 ± 0.75	0.50 ± 0.66	0.333	0.94 ± 0.67	0.55 ± 0.71	0.47 ± 0.67	0.37 ± 0.81	0.089
Fruits	1.20 ± 0.64	1.10 ± 0.50	0.125	1.20 ± 0.56	1.13 ± 0.58	1.17 ± 0.66	1.33 ± 0.54	0.525
Meat	**0.85 ± 0.85**	**0.21 ± 0.71**	** < 0.001**	**0.87 ± 0.69**	**0.57 ± 0.84**	**0.15 ± 0.77**	**0.71 ± 1.01**	**0.028**
Fish	0.48 ± 1.08	0.39 ± 0.99	0.459	0.08 ± 1.17	0.46 ± 1.04	0.60 ± 1.02	0.38 ± 0.96	0.485
Processed meat products	**0.75 ± 0.85**	**0.27 ± 0.94**	** < 0.001**	0.55 ± 0.74	0.49 ± 0.91	0.72 ± 0.95	0.73 ± 0.92	0.459
Fast foods	**0.91 ± 0.79**	**0.40 ± 0.91**	** < 0.001**	0.73 ± 0.53	0.71 ± 0.89	0.47 ± 1.01	0.67 ± 0.87	0.796
Dairy foods	**1.05 ± 0.75**	**0.81 ± 0.82**	**0.040**	0.95 ± 0.74	0.88 ± 0.78	1.07 ± 0.85	1.22 ± 0.76	0.188
Eggs	**0.84 ± 1.00**	**0.49 ± 1.22**	**0.041**	0.44 ± 1.22	0.69 ± 1.09	0.74 ± 1.17	0.61 ± 1.24	0.802
Snacks	**1.12 ± 0.64**	**0.85 ± 0.77**	**0.010**	1.17 ± 0.47	0.96 ± 0.70	0.98 ± 0.92	1.25 ± 0.61	0.176
Starchy staples & Beans	**0.79 ± 0.74**	**0.61 ± 0.63**	**0.028**	0.97 ± 0.73	0.71 ± 0.69	0.69 ± 0.70	0.51 ± 0.68	0.171

^a^differences between boys and girls by using Mann–Whitney U test (*P* < 0.05); ^b^differences among children with different weight status by using Kruskal–Wallis one-way ANOVA test. *P*-value < 0.05 are in bold type

The scores of instrumental feeding practice significantly differed among children with different weight status (*P* = 0.033), which was higher in parents with obese children compared to those with children with underweight (*P* = 0.026). No significant difference was found in eating behaviors among children with different weight status (all *P* > 0.050). Preferences for meats were variable among children with different weight status (*P* = 0.028).

Table 4 shows differences of parental feeding practices between fathers and mothers who responded to child’s daily diet and living, and completed the questionnaires of parental feeding practices. The use of emotional feeding was higher in mothers than fathers (*P* = 0.048). No difference was found in other parental feeding practices.

**Table 4:** Differences in parental feeding practices between fathers and mothers

	Fathers	Mothers	*P* value
Instrumental feeding	2.16 ± 0.77	2.38 ± 0.71	0.207
Encouragement	3.20 ± 0.50	3.31 ± 0.62	0.561
**Emotional feeding**	**2.18 ± 0.75**	**2.53 ± 0.69**	**0.048**
Control over eating	0.55 ± 0.49	0.70 ± 0.51	0.114

Mann–Whitney U tests were used to assess differences between mothers and fathers. *P*-value < 0.05 are in bold type

### Associations between parental feeding practices and children eating behaviors

Linear regression analysis was used to explore associations between parental feeding practices and children’s eating behaviors (Table 5). After the adjustment for confounding factors, parental emotional feeding practice was positively associated with emotional undereating among children (β 0.54, 95% CI 0.16 to 0.92, *P* = 0.006). There were other associations found between parental feeding practices and children’s eating behaviors.

**Table 5:** Associations between parental feeding practices and children’s eating behaviors by using linear regression analysis

	FR		EOE		EF		DD		SR		SE		EUE		FF	
	B 95% CI	*P*-value	B 95% CI	*P*-value	B 95% CI	*P*-value	B 95% CI	*P*-value	B 95% CI	*P*-value	B 95% CI	*P*-value	B 95% CI	*P*-value	B 95% CI	*P*-value
IFP	0.03 (-0.38,0.43)	0.900	-0.04 (-0.46,0.38)	0.845	-0.09 (-0.52,0.35)	0.695	0.21 (-0.23,0.65)	0.338	0.04 (-0.27,0.35)	0.808	0.03 (-0.35,0.41)	0.876	-0.25 (-0.63,0.13)	0.192	0.24 (-0.12,0.60)	0.191
EFP	0.12 (-0.29,0.53)	0.559	0.20 (-0.22,0.62)	0.336	0.29 (-0.14,0.73)	0.183	-0.13 (-0.56,0.31)	0.571	-0.10 (-0.42,0.21)	0.518	0.08 (-0.30,0.46)	0.674	**0.54** **(0.16,0.92)**	**0.006**	-0.23 (-0.59,0.14)	0.214
EEP	0.06 (-0.32,0.45)	0.749	0.13 (-0.27,0.53)	0.509	-0.20 (-0.61,0.22)	0.345	0.32 (-0.10,0.73)	0.133	0.29 (-0.01,0.59)	0.054	0.02 (-0.34,0.39)	0.907	-0.14 (-0.50,0.22)	0.437	0.15 (-0.20,0.49)	0.401
CEP	-0.01 (-0.47,0.46)	0.983	-0.26 (-0.74,0.23)	0.294	0.24 (-0.26,0.73)	0.351	-0.22 (-0.72,0.29)	0.389	-0.27 (-0.63,0.09)	0.139	-0.02 (-0.46,0.43)	0.946	0.05 (-0.39,0.49)	0.811	-0.16 (-0.58,0.26)	0.456

Adjusted for child’s age, sex and weight status, parental education and family incomes; *CI* confidence interval, *IFP* Instrumental feeding practice, *EFP* Emotional feeding practice, *EEP* Encouragement to eat practice, *CEP* Control over eating practice, *FR* Food responsiveness, *EOE* Emotional overeating, *EUE* Emotional undereating, *EF* Enjoyment of food, *DD* Desire to drink, *SR* Satiety responsiveness, *SE* Slowness in eating, *FF* Food fussiness. *P*-value < 0.05 are in bold type

### Associations between parental feeding practices and food preferences

In linear regression analyses, after the fully adjustment, instrumental feeding practice was found to negatively associate with preference for fish (β -0.47, 95% CI -0.94 to -0.01, *P* = 0.048) (Table 6). And parental encouragement feeding practice was positively lined to children’s liking for processed meat (β 0.43, 95% CI 0.08 to 0.77, *P* = 0.015) (Table 6). No significant association was found between other parental feeding practices and preferences for food groups.

**Table 6:** Associations between parental feeding practices and children’s food preferences by using linear regression analysis

	Vegetables		Fruits		Meats		Fish		Dairy foods		Eggs		Starchy staples & Beans		Processed meat products		Fast foods		Snacks	
	B 95% CI	*P*-value	B 95% CI	*P*-value	B 95% CI	*P*-value	B 95% CI	*P*-value	B 95% CI	*P*-value	B 95% CI	*P*-value	B 95% CI	*P*-value	B 95% CI	*P*-value	B 95% CI	*P*-value	B 95% CI	*P*-value
IFP	-0.24 (-0.57,0.09)	0.145	-0.08 (-0.35,0.18)	0.540	-0.19 (-0.58,0.21)	0.354	**-0.47** **(-0.94,-0.01)**	**0.048**	0.37 (-0.01,0.75)	0.054	-0.01 (-0.49,0.46)	0.957	0.02 (-0.33,0.38)	0.895	0.06 (-0.30,0.42)	0.734	0.26 (-0.15,0.66)	0.210	0.15 (-0.18,0.47)	0.379
EFP	0.11 (-0.23,0.45)	0.513	0.17 (-0.10,0.44)	0.213	0.18 (-0.22,0.58)	0.381	0.32 (-0.17,0.81)	0.201	-0.32 (-0.71,0.06)	0.096	-0.09 (-0.40,0.57)	0.724	-0.11 (-0.47,0.25)	0.538	-0.26 (-0.63,0.10)	0.157	-0.19 (-0.61,0.23)	0.361	-0.07 (-0.40,0.26)	0.661
EEP	-0.08 (-0.40,0.23)	0.600	-0.11 (-0.36,0.14)	0.388	0.10 (-0.28,0.48)	0.586	-0.06 (-0.51,0.39)	0.790	-0.12 (-0.48,0.25)	0.527	-0.04 (-0.50,0.42)	0.854	0.04 (-0.30,0.38)	0.814	**0.43** **(0.08,0.77)**	**0.015**	0.01 (-0.37,0.39)	0.971	-0.08 (-0.40,0.23)	0.598
CEP	-0.09 (-0.47,0.30)	0.658	0.01 (-0.30,0.31)	0.977	-0.07 (-0.53,0.39)	0.763	-0.10 (-0.64,0.45)	0.729	-0.02 (-0.46,0.42)	0.940	0.08 (-0.48,0.64)	0.787	0.05 (-0.36,0.47)	0.799	-0.22 (-0.64,0.20)	0.304	0.21 (-0.27,0.68)	0.384	-0.06 (-0.32,0.44)	0.750

Adjusted for child’s age, sex and weight status, parental education and family incomes; *CI* confidence interval, *IFP* Instrumental feeding practice, *EFP* Emotional feeding practice, *EEP* Encouragement to eat practice, *CEP* Control over eating practice. *P*-value < 0.05 are in bold type

## Discussion

This study mainly clarified individual parental feeding practices associated to eating behaviors and food preferences among Chinese children. Parents highly used encouragement to eating practice for their children. In addition, emotional feeding practice was greatly used by mothers than fathers who took care of kids’ daily diet and living. Importantly, parental emotional feeding practice was positively associated with children’s emotional undereating. Children with parents using higher level of encouragement to eat were likely to prefer processed meat products. There was a negative association between instrumental feeding practice and preference for fish. These findings extend current literature on specific parental feeding practices in relation to children’s eating behaviors and food preferences.

Parents who have boys in our study were likely to use control over eating practice compared to those have girls. It has also been shown that parents with daughters tended to use emotional feeding practice in South Asia, rather than those have sons [44]. It is indicated that the use of feeding practices by parent may also depend on children’s sex. Sex difference in parental control over eating in our study might be due to different controlling practices between sons and daughters by Chinese parents [45]. This can be explained to parenting style linked to parental feeding practice [46]. Furthermore, Sex difference of parental feeding practices amongst ethnicities might attribute to cultural variation. For instance, Chinese mothers are likely to use restrictive feeding for children, compared to White British, south Asian and Black mothers in the UK [47].

Our study revealed a higher level of use of emotional feeding by mothers than fathers. Previous studies found that fathers use more controlling practices than mothers [45, 48]. There was also no sex difference found in parental feeding practices in previous study [49]. These inconsistent results on use of feeding practices by mothers and fathers may be explained by diversity in study samples with different social or cultural traditions, as well as access to economic resources, which influence parent’s decision of using specific feeding practices [48, 50]. This might also explain the lower score of parental control over eating found in our study compared to previous study [22].

In the present study, results showed that boys obtained higher scores of food responsiveness, emotional overeating, enjoyment of food and desire to drink than girls. It has been shown that boys in five Thai primary public schools had higher degrees of enjoyment of food and desire to drink behaviors [51]. In addition, higher levels of food responsiveness and emotional overeating were found in teenage boys in the UK [52]. These findings may reveal a variety of sex differences in eating behaviors among children within different cultural backgrounds. Furthermore, there was a suggestion for inconsistent results that sex differences in some eating behaviors start to develop at some stage of children and adolescent [53]. It is supported with our findings that show significant sex differences in enjoyment of food, emotional overeating, desire to drink, food responsiveness and slowness in eating among older children. Similarly, there was no difference between boys and girls aged 1–6 years in Swedish [53].

Moreover, boys preferred meats, processed meats, fast foods, dairy foods, eggs, snacks and starchy staples & beans more than girls did in our study. Previous research has shown that boys had greater likings for fatty & sugary foods, meat, processed meat and eggs [54]. This has been explained to energy requirements by boys for an adaptive purpose [54]. In addition, girls disliking more foods may be due to weight and diet issues, probably because of social desirability influencing girls’ responding [55].

In the present study, we found that the use of instrumental feeding practice was different by parents whose children with different weight status, and its score was higher in parents with overweight and obese children compared to children with normal weight. Similarly, a prospective study showed that instrumental feeding practice predicts child BMI z-score one year later [56]. Previous study suggested that parents are more likely to use some feeding practices in response to their children’s weight, in turn influencing children’s body weight [57]. Furthermore, this is explained that instrumental feeding may lead to increased preference and consumption of high-calorie foods [56]. It is consistent with our results showing an association between instrumental feeding practice and preference for fish among children. We also found that preference for meats was higher in children with overweight than those with normal weight. These findings to some extent potentially indicate relationships of instrumental feeding practices to children’s food preferences and weight status.

In the present study, children were likely to have high degree of emotional undereating behavior when their parents had higher score of emotional feeding practice. Similar to previous findings that use of food by parents for emotional reasons is correlated with emotional undereating in 3–6 years old children [58]. This is explained that parents tend to use food to regulate child’s negative emotion, which is linked to loss of gut activity and less eating [59]. It is known that emotional undereating is a factor of reflecting ‘food avoid’ behaviors [60]. Therefore, emotional feeding practice should not be used for children to prevent the development of emotional undereating that is positively associated with obesity [61–63].

It has been demonstrated in our study that parental encouragement to eat was associated with children’s preference for processed meat products. Parental encouragement to eat practice refers to positive, gentle, supportive, and non-coercive practice to build on child’s healthy eating behaviors, and allow children to make decision [64]. It is suggested that repeated taste exposures can increase preferences for specific foods among children [65]. Previous research has found that encouragement to eat is positively related to child’s consumption of fruits and vegetables that are healthy foods [28]. Our finding might indicate that parents inaccurately perceived processed meats as a healthy food source of animal protein [66], and repeated to expose processed meats for their child. However, processed meats are unhealthy food because of excess energy [67, 68]. Thus, it is important for parents to build on correct perception of healthy foods when they repeat expose of specific foods to children for nutritional needs.

This study has some limitations to be discussed. First, the cross-sectional study limits conclusions about the direction of the associations found in this study. Future study would benefit from longitudinal designs to elucidate the direction of the associations of parental feeding practice with children’s eating behaviors, and food preferences. Second, it is important to note that the generalizability of the findings is limited by the small sample size. And data was collected from school-aged children in Shanghai, the most developed city in China. These findings therefore reflect samples within a high socio-economic status across China, and it is important to replicate this research with larger samples of more diverse socio-economic status to verify these findings. Third, some confounding cannot be included due to unmeasured varieties. The role of possible confounding factors such as parental age and BMI should also be examined. Fourth, food diaries or food frequency questionnaire were not used, which would limit us to provide additional strength to conclusions. Future research would benefit from using these questionnaires to make findings more widely applicable.

## Conclusion

This study identified associations of specific parental feeding practices with children’s eating behaviors and food preferences. In general, the current findings support the differences in the use of feeding practices regarding to parental and children’s sex. Importantly, parental emotional feeding practice was positively associated with children’s emotional undereating. The results also indicate that parental encouragement to eating was associated with preference for processed meat foods. In addition, there was a relationship of instrumental feeding practice to prefer fish. In view of these findings, it appears pertinent to further understand impacts of parental feeding practices on children’s healthy eating behaviors and preferences for healthy foods related to childhood weight issue.

## Supplementary Information

**Additional file 1.**

**Additional file 2.**

## Data Availability

The datasets used and analyzed during the current study are available from Min Hou, the project investigator, on reasonable request.

## Abbreviations

WGOC: Working Group for Obesity in China
PFPQ: Parental Feeding Practice Questionnaire
SD: Standard deviations
VIF: Variance inflation factors
CI: Confidence interval
