# Supplementary material for: Associations of parental feeding practices with children’s eating behaviors and food preferences: a Chinese cross-sectional study
Source: BMC Pediatr. 2023 Feb 18;23:84. doi: 10.1186/s12887-023-03848-y (PMC9938626; doi:10.1186/s12887-023-03848-y)
Supplement: Supplementary file 1 — Additional file 1. [file 12887_2023_3848_MOESM1_ESM.docx]

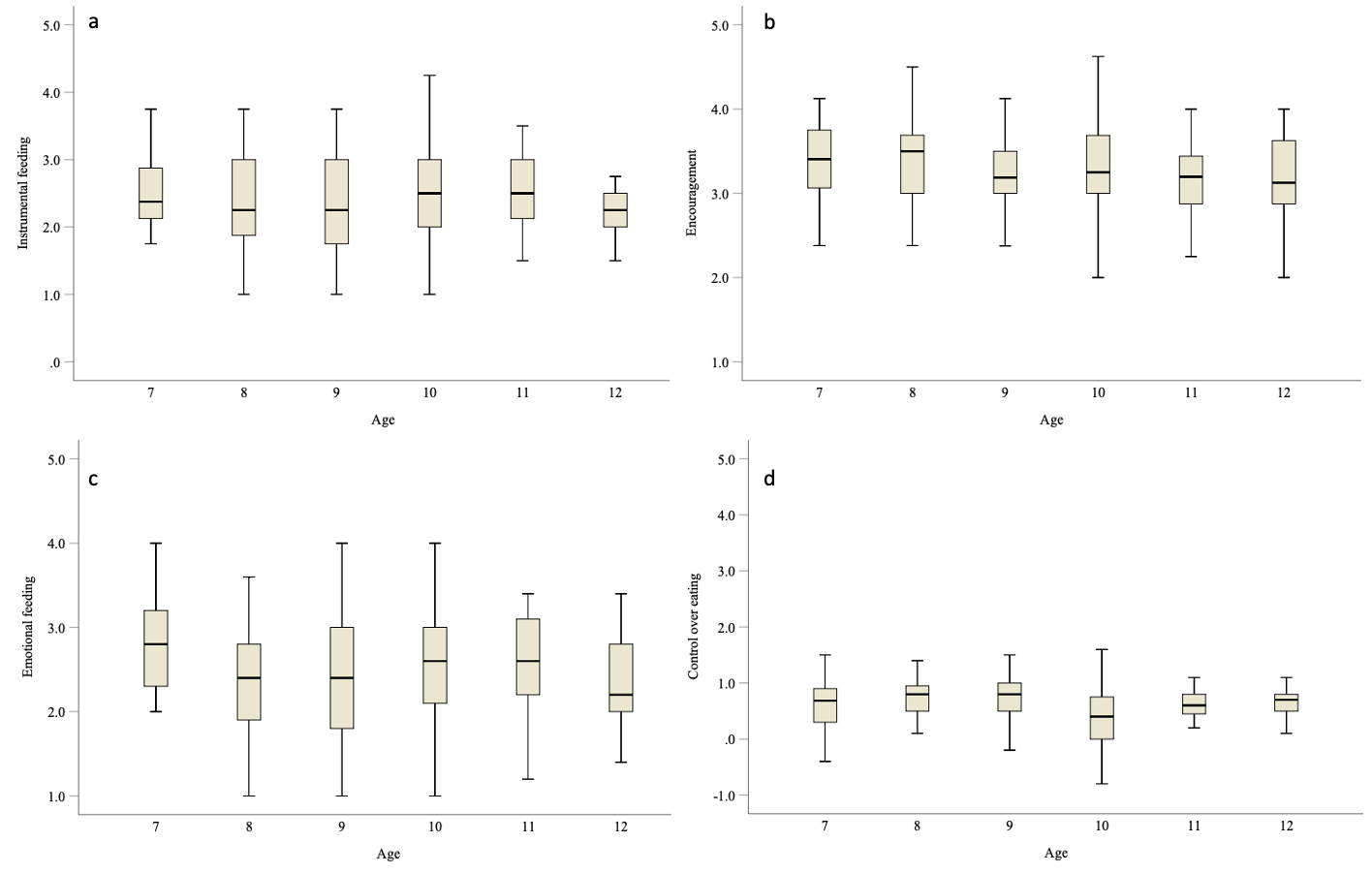


Figure S1 Parental feeding practices among children with ages

7 years old: n= 36, 8 years old: n= 62, 9 years old: n= 58, 10 years old: n= 46, 11 years old: n= 26, 12 years old: n= 15.

Differences among children with ages were analysed by using Kruskal-Wallis one-way ANOVA test.


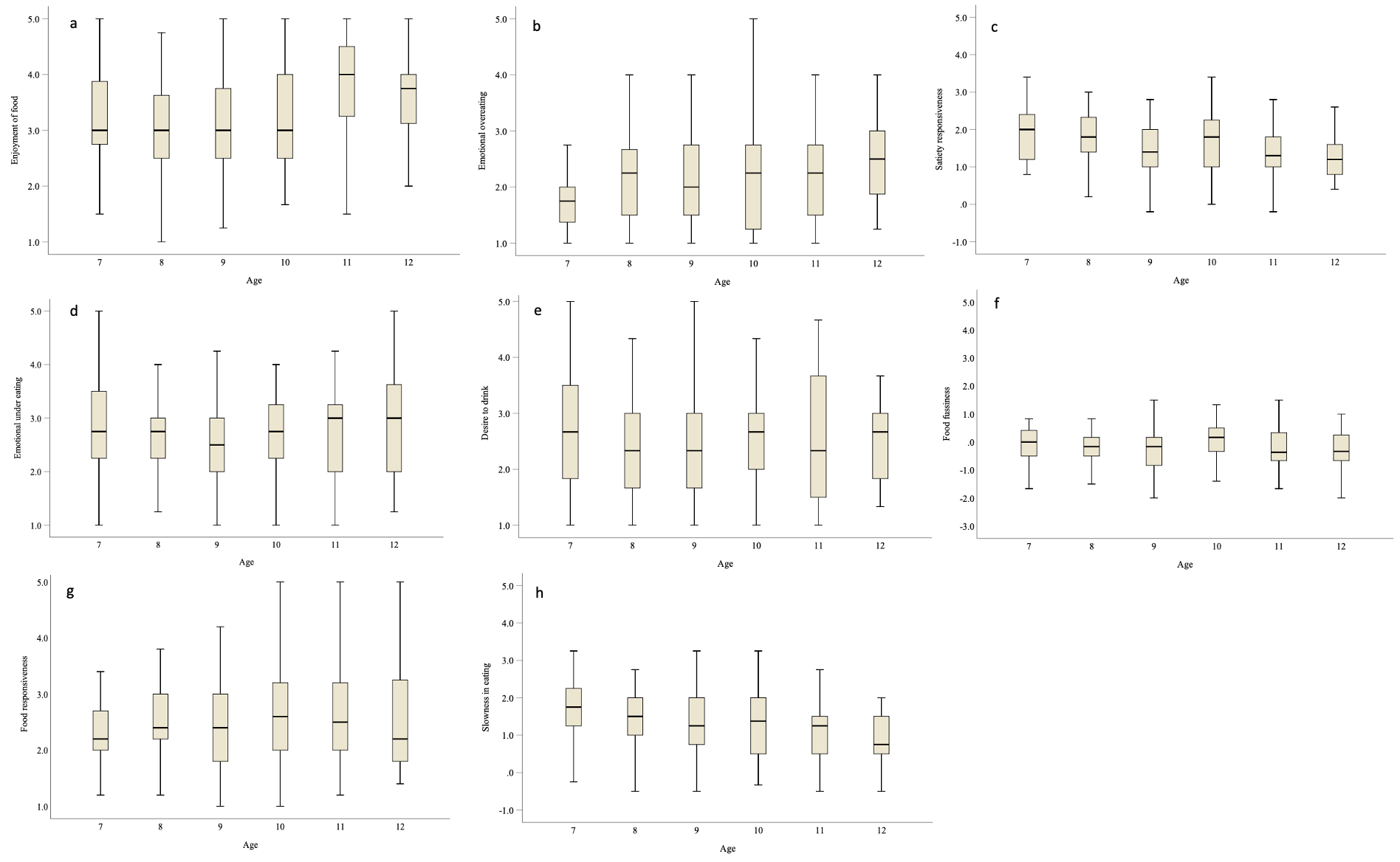


Figure S2 Eating behaviors among children with ages

7 years old: n= 36, 8 years old: n= 62, 9 years old: n= 58, 10 years old: n= 46, 11 years old: n= 26, 12 years old: n= 15.

Differences among children with ages were analysed by using Kruskal-Wallis one-way ANOVA test.


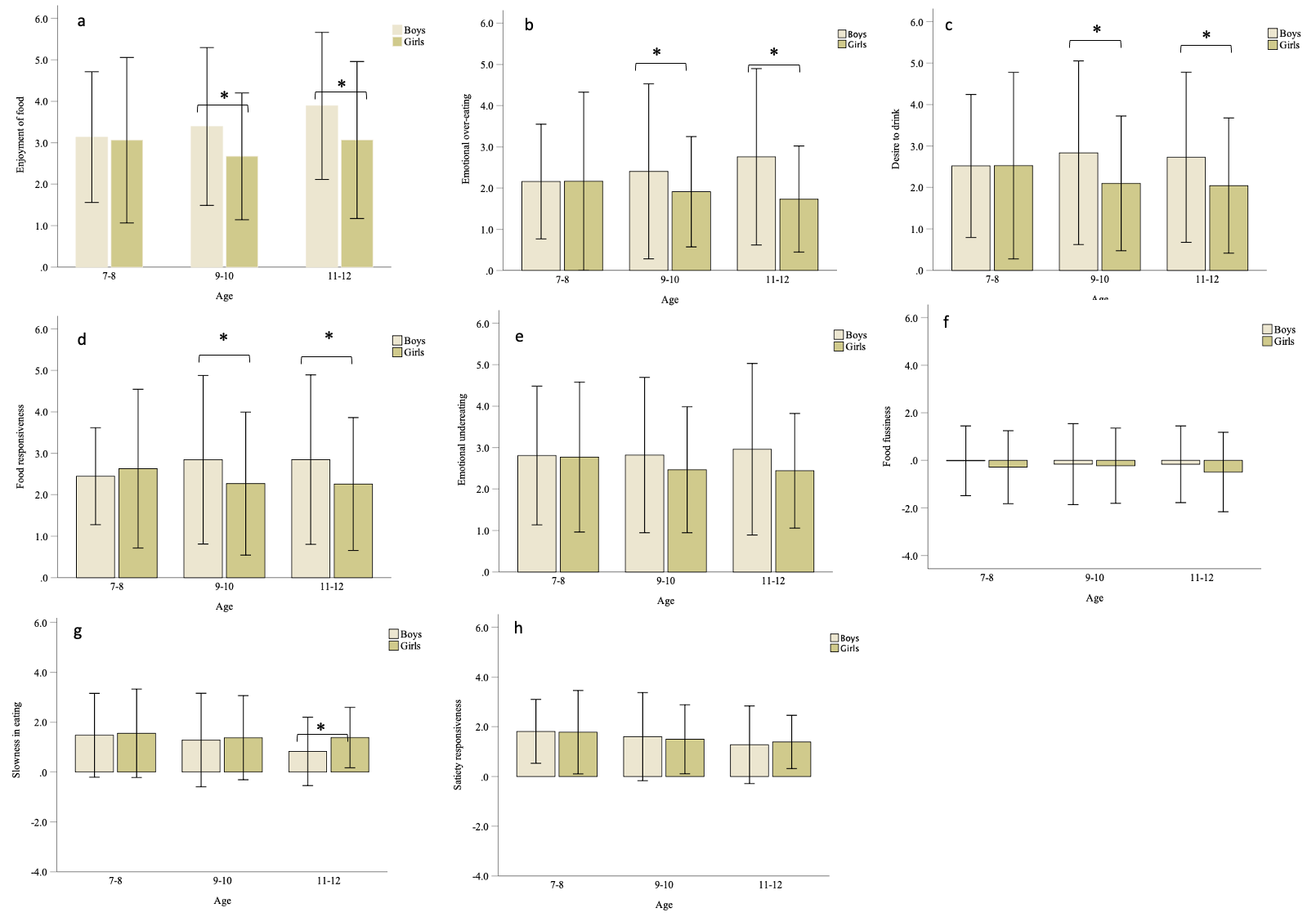


Figure S3 Differences in eating behaviors among children at different ages

Mann-Whitney U tests were used to assess differences between boys and girls. * as *P*-value < 0.05.
