# Supplementary material for: Associations of parental feeding practices with children’s eating behaviors and food preferences: a Chinese cross-sectional study
Source: BMC Pediatr. 2023 Feb 18;23:84. doi: 10.1186/s12887-023-03848-y (PMC9938626; doi:10.1186/s12887-023-03848-y)
Supplement: Supplementary file 2 — Additional file 2. [file 12887_2023_3848_MOESM2_ESM.docx]

**Table S1** Individual food preferences according to child’s sex and age

| Food item | Children who had tried food  (%) | Food preference score | P-value | |
| --- | --- | --- | --- | --- |
|  |  |  | Sex | Age |
| Vegetables (21) |  |  |  |  |
| Broccoli | 98.7 | 0.90±1.00 | 0.662 | 0.006 |
| Cabbage | 90.5 | 0.54±1.13 | 0.172 | 0.035 |
| Cauliflower | 98.3 | 0.82±0.99 | 0.337 | 0.153 |
| Green bean | 94.6 | 0.34±1.12 | 0.723 | 0.000 |
| Aubergine | 97.5 | 0.36±1.28 | 0.759 | 0.113 |
| Onion | 94.1 | 0.11±1.24 | 0.326 | 0.725 |
| White Turnip | 97 | 0.39±1.18 | 0.700 | 0.577 |
| Tomato | 96.6 | 0.85±1.08 | 0.956 | 0.495 |
| Cucumber | 99.2 | 0.87±0.98 | 0.383 | 0.418 |
| Carrot | 98.3 | 0.54±1.19 | 0.856 | 0.342 |
| Pepper | 84.9 | -0.36±1.37 | 0.000 | 0.079 |
| Green vegetables | 81.4 | 0.17±1.07 | 0.260 | 0.000 |
| Spinach | 98.3 | 0.6±1.14 | 0.157 | 0.036 |
| Chinese cabbage | 99.1 | 0.68±1.06 | 0.045 | 0.125 |
| Apium graveolens | 94.9 | 0.55±1.14 | 0.032 | 0.214 |
| Pumpkin | 97 | 0.52±1.13 | 0.948 | 0.032 |
| Asparagus lettuce | 93.2 | 0.65±1.1 | 0.172 | 0.117 |
| Lettuce | 96.2 | 0.61±1.15 | 0.552 | 0.010 |
| Chinese watermelon | 97.4 | 0.53±1.17 | 0.901 | 0.035 |
| Lotus root | 91.9 | 0.42±1.19 | 0.032 | 0.41 |
| Potato | 99.6 | 1.12±0.93 | 0.024 | 0.521 |
| Fruits (15) |  |  |  |  |
| Apple | 99.2 | 1.28±0.8 | 0.824 | 0.970 |
| Banana | 97 | 1.19±0.93 | 0.090 | 0.539 |
| Orange | 99.2 | 1.17±0.89 | 0.002 | 0.508 |
| Tangerine | 99.2 | 1.22±0.88 | 0.003 | 0.301 |
| Grape | 99.2 | 1.26±0.76 | 0.044 | 0.639 |
| Lemon | 89.3 | 0.44±1.24 | 0.562 | 0.015 |
| Peach | 98.7 | 1.34±0.79 | 0.642 | 0.691 |
| Pear | 99.6 | 1.28±0.83 | 0.372 | 0.340 |
| Plum | 84.9 | 0.78±1.05 | 0.033 | 0.124 |
| Strawberry | 97.9 | 1.38±0.87 | 0.508 | 0.393 |
| Water lemon | 99.6 | 1.42±0.78 | 0.078 | 0.416 |
| Mango | 97.4 | 1.24±0.93 | 0.442 | 0.610 |
| Kiwifruit | 97.9 | 0.89±1.08 | 0.667 | 0.097 |
| Pineapple | 96.2 | 0.82±1.09 | 0.456 | 0.241 |
| Honeydew | 98.3 | 1.15±0.97 | 0.298 | 0.164 |
| Meats (5) |  |  |  |  |
| Beef | 241(99.6) | 0.83±1.05 | 0.000 | 0.215 |
| Lamb | 95.4 | 0.35±1.2 | 0.000 | 0.018 |
| Pork | 98.3 | 0.38±1.22 | 0.000 | 0.011 |
| Chicken | 98.3 | 0.85±1.03 | 0.000 | 0.075 |
| Duck | 94.9 | 0.41±1.13 | 0.021 | 0.262 |
| Fish (2) |  |  |  |  |
| Fish | 98.3 | 0.31±1.22 | 0.114 | 0.094 |
| Prawn | 99.2 | 0.55±1.13 | 0.566 | 0.136 |
| Processed meat products (3) | |  |  |  |
| Bacon | 83.1 | -0.18±1.21 | 0.030 | 0.086 |
| Ham | 95.4 | 0.74±1.06 | 0.001 | 0.023 |
| Sausage | 97.5 | 0.91±1.05 | 0.000 | 0.103 |
| Fast food (4) |  | 0.68 ± 0.06 |  |  |
| Hansberger | 95.4 | 0.6±1.23 | 0.000 | 0.065 |
| Fired chicken | 97.9 | 0.9±1.14 | 0.000 | 0.481 |
| Fried fish | 90 | 0.1±1.26 | 0.016 | 0.584 |
| Chips | 98.3 | 1.01±1.1 | 0.009 | 0.118 |
| Dairy foods (3) |  |  |  |  |
| Yoghurt | 99.2 | 1.32±0.86 | 0.605 | 0.648 |
| Cheese | 87 | 0.62±1.14 | 0.060 | 0.295 |
| Cream | 98.3 | 0.70±1.20 | 0.058 | 0.468 |
| Eggs (2) |  | 0.68 ± 0.07 |  |  |
| Boiled eggs | 97.5 | 0.69±1.22 | 0.668 | 0.838 |
| Fried eggs | 96.1 | 0.65±1.18 | 0.056 | 0.256 |
| Snacks (11) |  |  |  |  |
| Salty snacks | 93.3 | 0.38±1.17 | 0.001 | 0.083 |
| Cookie | 99.2 | 0.87±1.09 | 0.007 | 0.065 |
| Chocolate biscuit | 99.6 | 0.99±1.06 | 0.016 | 0.892 |
| Shortcake | 96.2 | 0.44±1.3 | 0.062 | 0.727 |
| Cake | 96.6 | 1.18±0.97 | 0.013 | 0.251 |
| Icecream | 99.6 | 1.38±0.87 | 0.762 | 0.912 |
| Ice lolly | 99.6 | 1.38±0.85 | 0.731 | 0.905 |
| Dessert | 97.1 | 1.25±0.95 | 0.057 | 0.436 |
| Crisps | 99.2 | 1.2±0.95 | 0.011 | 0.774 |
| Preserved fruit | 89.5 | 0.64±1.1 | 0.732 | 0.293 |
| Sweets | 99.6 | 0.96±1.12 | 0.040 | 0.748 |
| Starchy staples &Beans (9) |  |  |  |  |
| White bread or steamed bun | 97 | 0.51±1.09 | 0.024 | 0.466 |
| Whole wheat bread | 91.7 | 0.58±1.08 | 0.053 | 0.083 |
| Oatmeal | 86.5 | 0.39±1.09 | 0.145 | 0.193 |
| Rice congee | 94.9 | 0.4±1.17 | 0.467 | 0.025 |
| Beans | 98.7 | 0.35±1.22 | 0.137 | 0.149 |
| Noodle | 99.2 | 0.94±1.04 | 0.236 | 0.545 |
| Sweet potato | 97.9 | 0.81±1.09 | 0.460 | 0.092 |
| Corn | 97.1 | 1.06±0.93 | 0.981 | 0.254 |
| Rice | 98.7 | 1.04±1.07 | 0.032 | 0.144 |
| Ungrouped foods |  |  |  |  |
| Canned fish | 55 | -0.14±1.04 | 0.174 | 0.828 |
| Mushroom | 98.7 | 0.68±1.21 | 0.394 | 0.083 |
| Kelp | 98.3 | 0.54±1.25 | 0.253 | 0.002 |
| Butter | 82.9 | 0.35±1.2 | 0.031 | 0.268 |
